# Intracorporeal reinforcement with barbed suture is associated with low anastomotic leakage rates after laparoscopic low anterior resection for rectal cancer: a retrospective study

**DOI:** 10.1186/s12893-022-01782-x

**Published:** 2022-09-09

**Authors:** Haiping Lin, Minhao Yu, Guangyao Ye, Shaolan Qin, Hongsheng Fang, Ran Jing, Tingyue Gong, Yang Luo, Ming Zhong

**Affiliations:** Department of Gastrointestinal Surgery, RenJi Hospital, School of Medicine, Shanghai Jiao Tong University, Shanghai, 200127 China

**Keywords:** Rectal cancer, Intracorporeal reinforcement, Barbed suture, Anastomotic leakage, Laparoscopic low anterior resection

## Abstract

**Background:**

Anastomotic leakage (AL) is one of most severe postoperative complications following low anterior resection (LAR) for rectal cancer, and has an adverse impact on postoperative recovery. The occurence of AL is associated with several factors, while few studies explored the role of intracorporeal barbed suture reinforcement in it.

**Methods:**

Consecutive cases underwent laparoscopic LAR for rectal cancer from Mar. 2018 to Feb. 2021 in our center were retrospectively collected. Cases were classified into the intracorporeal barbed suture reinforcement group and the control group according to whether performing intracorporeal reinforcement with barbed suture, and AL incidences were compared between two groups. Propensity score matching (PSM) was then performed based on identified risk factors to reduce biases from covariates between two groups. AL incidences in the matched cohort were compared.

**Results:**

A total of 292 cases entered into the study, and AL incidences were significantly lower in the intracorporeal barbed suture reinforcement group compared with the control group (10.00% vs 2.82%, *P* = 0.024). Sex, BMI, preoperative adjuvant chemoradiotherapy and anastomotic level were chose for PSM analyses based on previous studies. In the matched cohort, the AL incidences were still significantly lower in the intracorporeal barbed suture reinforcement group (10.57% vs 2.44%, SD = 0.334).

**Conclusions:**

Intracorporeal barbed suture reinforcement is associated with low AL incidences after laparoscopic LAR for rectal cancer, which is a potential procedure for reducing AL and worthy of application clinically.

**Supplementary Information:**

The online version contains supplementary material available at 10.1186/s12893-022-01782-x.

## Background

For rectal cancer patients, long term oncological outcomes for open versus laparoscopic surgery are comparable, while laparoscopic surgery is associated with advantages of minimal trauma and quick recovery, thus laparoscopic rectal resection is widely applied in clinical settings[1, 2]. However, laparoscopic surgery is helpless to reduce incidences of anastomotic leakage (AL), one of most severe postoperative complications of rectal cancer surgery[3–5]. Studies have shown that the occurrence of AL will extend postoperative hospital stay and postpone receiving postoperative adjuvant chemoradiotherapy[6, 7]. Moreover, AL has an adverse impact on postoperative life qualities, and will significantly increase local recurrences and mortalities[8–11]. Consideing these AL-associated adverse effects, reducing postoperative AL incidence has become a critical issue for clinicians.

The occurence of AL is associated with several factors, such as anastomosis level, anastomotic blood supply, anastomotic techniques, anastomotic tension, enteric pressure and reinforcing materials[12–14]. Previous studies have reported that the formation of “dog ears” area, a spot formed on bilateral intersecting margins at the distal rectal stump, is a weak anastomotic tissue that lacks blood supply and tends to develop inflammatory edema, resulting in a dangerous area for postoperative AL [15, 16]. Therefore, surgeons tend to reinforce anastomotic site, especially the “dog ears” area, to reduce postoperative AL [17]. However, few studies have until now compared postoperative AL incidences in patients receiving anastomotic reinforcement with those who not.

In addition, reinforcing materials has updated a lot in recent years, and the upgraded materials are supposed to reduce AL. Barbed suture is a type of knotless surgical suture that has barbs on its surface. As early as 1960s, some researchers tried to design the barbed suture, while the research was restricted due to the limitation of material and technology [18]. In recent years, absorbable unidirectional barbed suture, with a uniform distribution of small barbs on the surface, develops as a new type of surgical suture, of which the unidirectional zigzag structure is circularly distributed on the suture surface to ensure that tissues can be connected closely and seamlessly. There are 20 microstrips per centimeter on the suture to ensure the firmness of the suture, and a self-anchoring ring at the end of the suture. Therefore, this unidirectional barbed suture has the advantages of no need for knotting, shorter suture time and reduced intraoperative bleeding. Previous studies have demonstrated the efficiency of barbed suture in other surgeries, such as gynecological surgeries, biliary surgeries and right colectomy, while, to our knowledge, no study has reported its application in laparoscopic rectal surgery [19].

Taken these into consideration, we retrospectively collected consecutive cases with laparoscopic low anterior resection (LAR) for rectal cancer, and compared incidence rates of AL between those treated with intracorporeal barbed suture reinforcement and those not, aiming to demonstrate that intracorporeal barbed suture reinforcement is associated with low AL incidences after laparoscopic LAR for rectal cancer.

## Methods and materials

### Inclusion and exclusion criteria

From Mar. 2018 to Feb. 2021, consecutive cases of patients with rectal cancer who underwent laparoscopic LAR at the Department of colorectal surgery, Renji Hospital, Shanghai Jiao Tong University School of Medicine were retrospectively collected. This study was approved by the Ethics Committee of of the Renji Hospital, Shanghai Jiao Tong University School of Medicine.

**Fig. 1:**
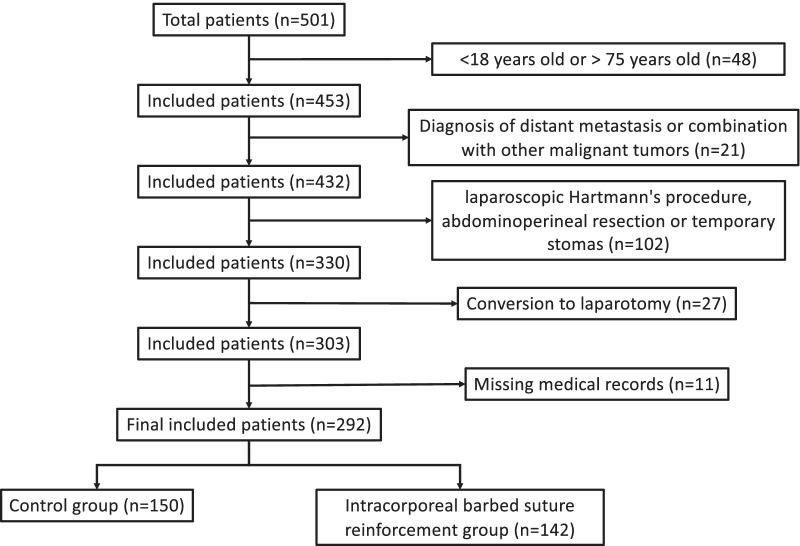
Flowchart showing selection processes

The inclusion criteria were (1) cases diagnosed as rectal cancer according to preoperative endoscopy and postoperative pathological reports; (2) cases underwent laparoscopic LAR for rectal cancer for the first time; (3) cases underwent sigmoid-rectal end-to-end anastomosis and distance of colorectal anastomosis from the anal verge ranged from 1.0 to 5.0 cm; (4) cases with age of 18–75 years old. The exclusion criteria were (1) cases diagnosed of distant metastasis or combined with other malignant tumors according to preoperative examinations, such as computed tomography (CT) and magnetic resonance imaging; (2) cases underwent laparoscopic Hartmann's procedure and abdominoperineal resection; (3) cases suffered a conversion to laparotomy; (4) cases underwent protective stomas when there was positive leak test, obvious anastomotic tension or no enough anastomotic blood supply (juded by indocyanine green (ICG) fluorescence imaging analyses); (5) cases without intact medical records. The selection process was summarized as a flowchart in Fig. 1.

### Surgical and follow-up procedures

Preoperative preparations, such as bowel preparation, for all patients were same, and surgical procedures were performed by the same surgical group. Patients were placed in lithotomy- Trendelenburg position. Five ports were placed generally, and a pneumoperitoneum of 12–14 mmHg was maintained during the surgery. All surgical procedures were performed according to the "radical surgery" principle. The rectum dissociation and lymph nodes dissection were conducted laparoscopically, and the distal rectum was transected 2–4 cm below the tumor margin; the circular stapler was used to finish the sigmoid-rectal end-to-end anastomosis.

All surgeries were performed by one surgical team (MZ as the surgeon, MY and YL as the assistants). Prior to decide whether or not to perform protective stoma, anastomotic tension was judged by the surgeon, and leak tests and indocyanine green (ICG) fluorescence imaging analyses were conducted routinely. When there was obvious anastomotic tension, positove leak test or positive ICG fluorescence imaging analysis, a protective stoma was placed and the patient will not be included into our retrospective collection.

The intracorporeal reinforcement was performed based on year, that is, cases from Mar. 2018 to Aug. 2019 underwent no intracorporeal reinforcement and cases from Sept. 2019 to Feb. 2021 underwent intracorporeal reinforcement. The surgeon, a colorectal specialist and chief physician with over 15 years of laparoscopic colorectal resection experience, has accomplished the learning curve, therefore time-biases were minor in the study. In the intracorporeal barbed suture reinforcement group, V-LOC™ barbed suture (3-0) (COVIDIEN, Beijing, China) was used for continuous suture to close the gap between mesocolon and mesorectum, and strengthen the "dog ears" area, the intersection area between linear cutting lines for transecting rectum and circular cutting lines for sigmoid-rectal end-to-end anastomosis (Additional file 1); in the control group, no intracorporeal reinforcing suture was performed. The negative pressure drainage tube was inserted into the abdominal cavity, and the transanal drainage tube was placed for decompression in all cases. Other surgical procedures and perioperative managements remained same between the two groups.

The follow-up procedures were mainly based on the NCCN guidelines, including medical history, physical examination, cancer biomarkers, chest and abdominal/pelvis CT/MRI. In addition, colonoscopy was performed 6 months after surgery to monitor the relapse and stenosis status.

### Data collection

The collected data included baseline characteristics [age, sex, body mass index (BMI), American Society of Anesthesiologists (ASA) status, etc.], operative recordings (surgical time, estimated blood loss, anastomotic level, etc.), and postoperative data (AL incidence rates, ventilation time). The definition of AL was referred to the proposal of the International Study Group of Rectal Cancer (ISREC), that is clinical symptoms of leakage (fever or abdominal pain), pelvic abscess confirmed by CT, water-soluble contrast enema or endoscope, or fecal discharge or pus from drainage tubes. Considering subclinical (grade A according to the ISREC defination) AL is hard to detect and has no significant influence to postoperative recovery, our study only focused on clinical (grade B/C according to the ISREC defination) AL. Considering the existence of delayed ALs, which mainly happens to patients with a diverting stoma, we further defined the diagnosis of no AL in patients without diverting stoma as no AL signs in both clinical symptoms and imagings, and taking semifluid at least 3 days without AL symptoms before hospital discharge[20].

According to the preoperative hemoglobin level (110.0 g/L in female and 120.0 g/L in male), anemia status was classified into anemia or not. According to the preoperative albumin level (35.0 g/L), albumin status was classified into low or normal status. The anastomotic level was defined as the distance from anastomotic site to verge of anal.

### Statistical analysis

To reduce biases from covariates and achieve balance between the intracorporeal barbed suture reinforcement group and control group, a propensity score matching (PSM) was conducted[21, 22]. Four variables, including sex, BMI, preoperative adjuvant chemoradiotherapy and anastomotic level, were selected for PSM based on previous studies [4, 12, 13, 23–30]. Based on the four variables, cases were matched with a 1:1 ratio using the nearest neighbor method with a caliper value of 0.1. Balances between the intracorporeal barbed suture reinforcement and control groups in matched cohort were evaluated by standard differences (SDs) with < 0.2 as an appropriate balancing. Then AL incidence rates between matched intracorporeal barbed suture reinforcement group and control group were compared.

Continuous variables were presented as medians with standard differences. For continuous variables, depending on whether variables were normally distributed or not, the Student’s t test or Wilcoxon rank-sum test was used as appropriate for intergroup comparisons between two groups and the Analysis of variance (ANOVA) or Kruskal–Wallis rank-sum test for intergroup comparisons was used among multiple groups. For categorical variables, the Chi-square test or Fisher exact test was used for intergroup comparisons.

All statistical analyses were conducted using the R software (version 4.0.3) and *P* < 0.05 was considered to be statistically significant.

## Results

### Baseline characteristics

There was a total of 501 rectal cancer cases underwent laparoscopic LAR by our surgical team from Mar 2018 to Feb 2021. Among them, 48 cases were excluded for age 18 or younger or 75 or older; 21 cases were excluded for diagnosis of distant metastasis or combination with other malignant tumors; 102 cases were excluded for undergoing laparoscopic Hartmann's procedure, abdominoperineal resection or temporary stomas; 27 cases were excluded for conversion to laparotomy; 11 cases were excluded for missing medical records. Finally, 292 rectal cancer cases entered into the study, including 157 males and 135 females (Fig. 1). The median age of the cohort was 64 years old, and the median BMI was 22.04 kg/m^2^. There were 22 cases staged as stage I, 105 cases as stage II and 165 cases as stage III according to the AJCC staging.

Patients were divided into the intracorporeal barbed suture reinforcement group (n = 142) and control group (n = 150) according to whether intracorporeal barbed suture reinforcement was conducted. Baseline characteristics of the two groups were summarized in Table 1.

**Table 1:** Baseline characteristics, intraoperative and postoperative outcomes between the intracorporeal barbed suture reinforcement and control groups

	Intracorporeal barbed suture reinforcement group n = 142	Control group n = 150
Age (year)	65.00 (7.53)	59.50 (10.57)
Sex		
Male	79 (55.63%)	78 (52.00%)
Female	63 (44.37%)	72 (48.00%)
BMI (kg/m^2^)	22.28 (2.85)	21.41 (2.89)
ASA		
Grade 1	70 (49.30%)	93 (62.00%)
Grade 2	70 (49.30%)	55 (36.67%)
Grade 3	2 (1.41%)	2 (1.33%)
Smoking		
Yes	39 (27.46%)	37 (24.67%)
No	103 (72.54%)	113 (75.33%)
Diabetes		
Yes	21 (14.79%)	14 (9.33%)
No	121 (85.21%)	136 (90.67%)
Anemia		
Yes	17 (11.97%)	17 (11.33%)
No	125 (88.03%)	133 (88.67%)
Preoperative albumin level		
Normal	138 (97.18%)	146 (97.33%)
Low	4 (2.82%)	4 (2.67%)
Preoperative adjuvant chemoradiotherapy		
Yes	18 (12.68%)	15 (10.00%)
No	124 (87.32%)	135 (90.00%)
Previous abdominal surgery history		
Yes	7 (4.93%)	6 (4.00%)
No	135 (95.07%)	144 (96.00%)
Tumor size (cm)	3.75 (1.48)	4.00 (1.57)
Stage		
Stage I	13 (9.15%)	9 (6.00%)
Stage II	48 (33.80%)	57 (38.00%)
Stage III	81 (57.04%)	84 (56.00%)
Tumor distance (cm)	7.00 (1.18)	7.00 (1.27)
Stapler firings		
< 3	133 (93.66%)	142 (94.67%)
≥ 3	9 (6.34%)	8 (5.33%)
Reserve of LCA		
Yes	86 (60.56%)	89 (59.33%)
No	56 (39.44%)	61 (40.67%)
Anastomosis level (cm)	3.00 (1.10)	3.00 (1,19)
Number of retrieved LNs	17.00 (4.62)	16.50 (3.88)
Surgical time (min)	147.50 (35.05)	130.00 (32.07)
Estimated blood loss (ml)	100.00 (73.96)	100.00 (75.01)
Postoperative ventilation time (h)	60.00 (20.03)	72.00 (19.74)
AL		
Yes	4 (2.82%)	15 (10.00%)
No	138 (97.18%)	135 (90.00%)
Hospital stay (day)	7.50 (3.18)	8.00 (4.12)

*BMI* body mass index, *ASA* American Society of Anesthesiologist, *CEA* carcinoma embryonic antigen, *LCA* left colic artery, *LN* lymph node, *AL* anastomotic leakage

### Intraoperative and postoperative outcomes

**Fig. 2:**
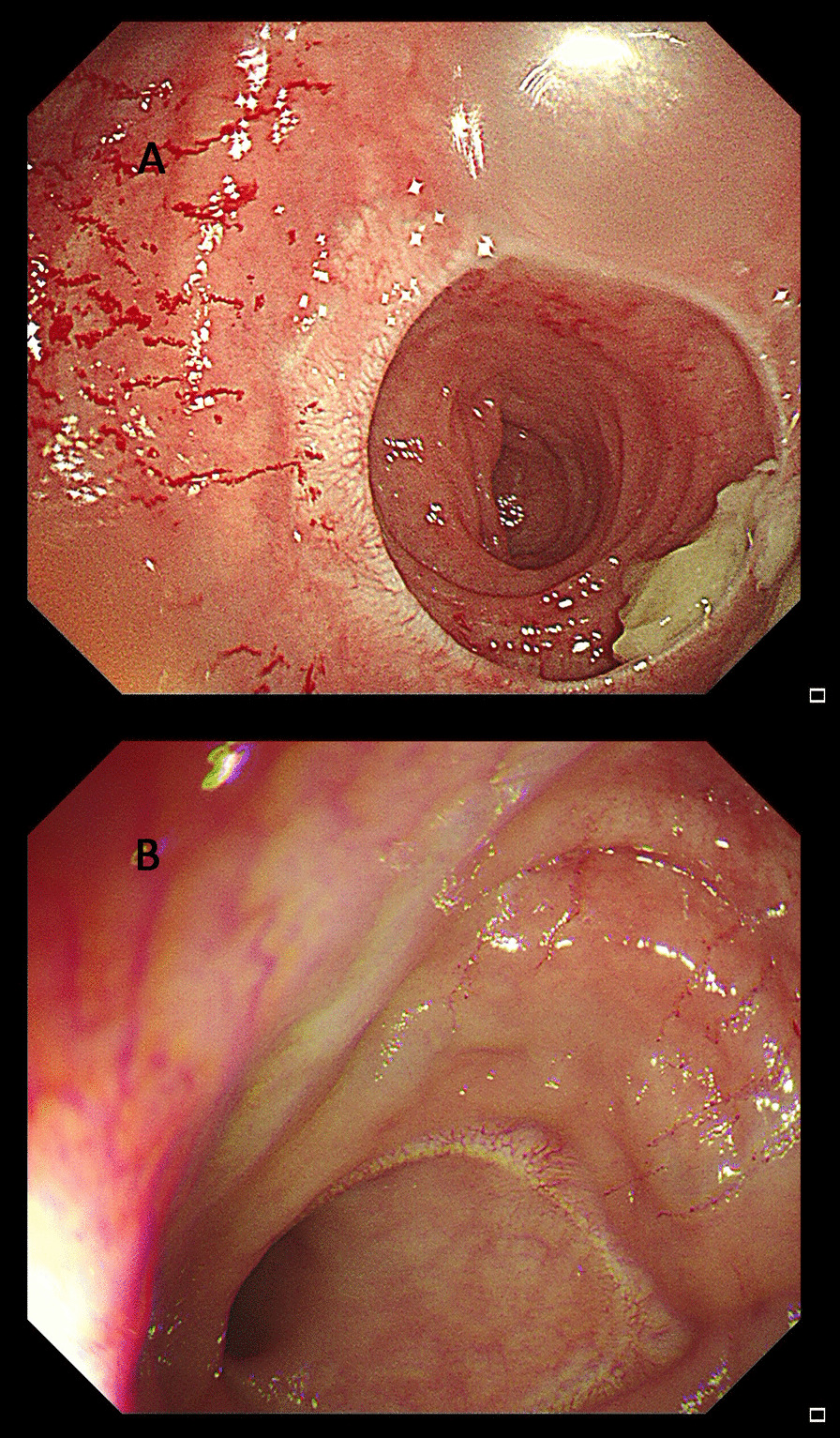
Anastomosis status examined by colonoscopy 6 months after surgery in (**A**) the intracorporeal barbed suture reinforcement group and (**B**) the control group

Table 1 showed that the surgical time in the intracorporeal barbed suture reinforcement group was significantly longer than that in the control group (*P* < 0.001), while there were no differences in number of retrieved lymph nodes (LNs) number (*P* = 0.327) and estimated blood loss (*P* = 0.326) between the two groups. In addition, no conversion to laparotomy operation happened in both two groups, an there were no differences in postoperative ventilation time (*P* = 0.099) and hospital stay (*P* = 0.126) between the two groups. As for the AL incidence, the incidence rates of AL in the intracorporeal barbed suture reinforcement group were significantly lower than that in the control group (2.82% vs 10.00%, *P* = 0.024). Most AL (15 of 19) were grade B and only 4 AL were grade C (2 in the intracorporeal barbed suture reinforcement group and 2 in the control group). Furthermore, no anastomotic stricture was observed in both intracorporeal barbed suture reinforcement and control groups, which is examined by colonoscopy six months after surgery (Fig. 2).

### Comparisons in the matched cohort

Considering there were only 19 AL events in our study, we chose 4 covariates (5 events-per-variable) for PSM analyses according to rule of thumb for PSM. Sex, preoperative adjuvant chemoradiotherapy, anastomotic level and surgical time were then selected to be covariates for propensity score matching, and there were 123 intracorporeal barbed suture reinforcement cases and 123 control cases in the matched cohort. Baseline characteristics and tumor-related factors became more comparable between the two types of cases except for age (SD = 0.642), ASA status (SD = 0.232) and diabetes (SD = 0.222), demonstrating covariates biases were reduced and balance was almost achieved between the two groups (Table 2).

**Table 2:** Baseline characteristics, intraoperative and postoperative outcomes between the intracorporeal barbed suture reinforcement and control groups in matched patients

	Intracorporeal barbed suture reinforcement group n = 123	Control group n = 123	Standardized difference
Age (year)	65.00 (6.71)	60.00 (10.70)	0.642
Sex			0.033
Male	67 (54.47%)	65 (52.85%)	
Female	56 (45.53%)	58 (47.15%)	
BMI (kg/m^2^)	22.32 (2.74)	21.51 (2.77)	0.059
ASA			0.232
Grade 1	61 (49.59%)	75 (60.98%)	
Grade 2	61 (49.59%)	47 (38.21%)	
Grade 3	1 (0.81%)	1 (0.81%)	
Smoking			< 0.001
Yes	32 (26.02%)	32 (26.02%)	
No	91 (73.98%)	91 (73.98%)	
Diabetes			0.222
Yes	20 (16.26%)	11 (8.94%)	
No	103 (83.74%)	112 (91.06%)	
Anemia			0.053
Yes	14 (11.38%)	12 (9.76%)	
No	109 (88.62%)	111 (90.24%)	
Preoperative albumin level			< 0.001
Normal	120 (97.56%)	120 (97.56%)	
Low	3 (2.44%)	3 (2.44%)	
Preoperative adjuvant chemoradiotherapy			0.028
Yes	11 (8.94%)	12 (9.76%)	
No	112 (91.06%)	111 (90.24%)	
Previous abdominal surgery history			0.039
Yes	5 (4.07%)	6 (4.88%)	
No	118 (95.93%)	117 (95.12%)	
Tumor size (cm)	4.00 (1.46)	4.00 (1.63)	0.164
Stage			0.098
Stage I	10 (8.13%)	7 (5.69%)	
Stage II	41 (33.33%)	43 (34.96%)	
Stage III	72 (58.54%)	73 (59.35%)	
Tumor distance (cm)	7.00 (1.14)	7.00 (1.23)	0.033
Stapler firings			< 0.001
< 3	115 (93.50%)	115 (93.50%)	
≥ 3	8 (6.50%)	8 (6.50%)	
Reserve of LCA			0.117
Yes	79 (64.23%)	72 (58.54%)	
No	44 (35.77%)	51 (41.46%)	
Anastomosis level (cm)	3.00 (1.10)	3.00 (1.18)	0.043
Number of retrieved LNs	18.00 (4.83)	17.00 (3.79)	0.224
Surgical time (min)	150.00 (33.12)	130.00 (30.46)	0.576
Estimated blood loss (ml)	100.00 (69.15)	100.00 (74.56)	0.027
Postoperative ventilation time (h)	72.00 (19.78)	72.00 (19.76)	0.137
AL			0.334
Yes	3 (2.44%)	13 (10.57%)	
No	120 (97.56%)	110 (89.43%)	
Hospital stay (day)	7.00 (2.32)	8.00 (4.14)	0.313

*BMI* body mass index, *ASA* American Society of Anesthesiologist, *CEA* carcinoma embryonic antigen, *LCA* left colic artery, *LN* lymph node, *AL* anastomotic leakage

In the matched cohort, compared with the control group, there was more retrieved LNs (SD = 0.224), longer surgical time (SD = 0.576) and shorter hospital stay (SD = 0.313) in the intracorporeal barbed suture reinforcement group, while no differences were observed in estimated blood loss (SD = 0.027) and postoperative ventilation time (SD = 0.137). Most importantly, the AL incidence rates were still significantly lower in the intracorporeal barbed suture reinforcement group (2.44% vs 10.57%, SD = 0.334) (Table 2).

## Discussion

AL, which means a leak of luminal contents from a defected anastomotic site into the abdominal cavity, is one of the most severe postoperative complications following rectal cancer surgery, and it will induce intra-abdominal infections and pelvic abscess, influencing postoperative life quality and prognosis [8–11]. In our study, through retrospectively analyzing 292 consecutive cases with laparoscopic LAR for rectal cancer, we found the overall AL incidence rate was 6.51%, which was consistent with rates of 3%-21% in previous studies [7, 28, 31–34]. The AL incidence rates were 10.00% in the control group and 2.82% in the barbed suture group, suggesting the combination of using barbed suture, strengthening of “dog ears” area and closing mesocolon-mesorectum gaps is associated with low AL incidence rates.

Barbed suture, as a novel type of suture material, have been widely used in other surgeries [35–39]. In gynecological surgery, Angioli R et al. found the barbed suture helped to close incisions of uterus rapidly and reliably, reduced suture time and intraoperative bleeding, and decreased surgical difficulties significantly [35]. In laparoscopic general surgery, the barbed suture was also used widely for closing the mesentery and abdominal wall, as well as primary closure for laparoscopic common bile duct exploration [37]. There were also studies regarding applications of barbed suture in right colectomy, showing it efficiency in reducing both intraoperative bleedings and postoperative leaks, while no study has reported its application in rectal cancer surgery [36].

As metioned in the Background part, "dog ears" areas are two stapled corners of the rectal stump formed by linear transection of rectum, and are the potential ischemic areas of AL [15, 16]. In addition, a gap is likely to be formed between mesocolon and mesorectum when performing colon-rectal end-to-end anastomosis in rectal cancer surgery, causing delayed healing postoperatively and increasing risks of AL. Therefore, we speculate that, according to results of our study, reinforcing the "dog ears" area and closing the gap with barbed suture is likely to reduce anastomotic tension, improve regional blood supply, and avoid fissures in the anastomotic sites, all of which may contribute to reducing incidences of postoperative AL.

Previously, few studies have focused on the role of reinforcing sutures in reducing AL incidences following laparoscopic rectal cancer surgery [40–42]. Gadiot et al. first proposed that three or four sutures antitraction suturing at the circular end-to-end anastomosis was able to reduce anastomotic failure, with 1% in the sutured group and 11% in the control group [40]. However, the study only included 126 patients in total, which restricted its statistical reliability. Maeda et al. then reported that intracorporeal reinforcing sutures was useful for reducing AL rates in a high-risk group (a tumor site from the anal verge of ≤ 5 cm or tumor size of ≥ 4 cm) while not in a low-risk group [41]. In their study, the time span was more than 6 years, and most of the reinforcing sutures cases belonged to the late period, which indicated that laparoscopic surgical skills may have an impact on the AL rates.

Different from previous studies, our study only focused on rectal cancer patients underwent laparoscopic LAR with distance of colorectal anastomosis from the anal verge ranged from 1.0 to 5.0 cm. In addition, we included nearly three hundred patients within 3 years, and all surgical procedures were performed by the same surgical team in our study to reduce influences from laparoscopic surgical skills. Furthermore, the PSM analyses were conducted to make the intracorporeal barbed suture reinforcement group and control group more comparable, which increased the credibility of our results that intracorporeal barbed suture reinforcement is likely to be an efficient way for preventing AL.

In addition, we found several tips is helpful for reducing suturing time and enhancing anastomotic strengthen. When suturing anti-mesenteric borders, inserting the needle approximately 0.5 cm from the anastomotic line and keeping needle distances of 1.0 cm are appropriate; when suturing mesenteric borders, suturing proximal mesocolon and distal mesorectum, forming a cover for covering anastomotic sites, especially the posterior rectal wall.

However, there are several limitations in our study. The study is a single-center retrospective study, and potential biases such as selection biases are inevitable in the study. There are also some confounding variables between the two groups in our study, so we perform PSM analyses to adjust for confounding, attempting to estimate causal effects between intracorporeal barbed suture reinforcement and AL, while conclusions from our retrospective study wait for further confirmation of randomized trials [43]. In addition, cases included into the study is relatively limited, although our study has the largest sample size in studies regarding intracorporeal barbed suture reinforcement to date. Therefore, we hope randomized controlled trials can be conducted in future to further demonstrate the efficacy of intracorporeal barbed suture reinforcement.

## Conclusion

In conclusion, our study found intracorporeal barbed suture reinforcement was associated with low incidences of AL after laparoscopic low anterior resection for rectal cancer.

## Supplementary Information

**Additional file 1:** An example of intracorporeal reinforcement with barbed suture following low anterior resection for rectal cancer.

## Data Availability

The datasets used and/or analyzed during the current study are available from the corresponding author on reasonable request.
